# Mortality and Insanity in Separate Plan Prisons in England and America
*Read by Dr. Parrish at a meeting of the College of Physicians of Philadelphia, Sept. 2nd, 1851.


**Published:** 1852-10-01

**Authors:** 


### MORTALITY AND INSANITY IN SEPARATE PLAN PRISONS IN ENGLAND AND AMERICA.*

(*Concluded from page* 406.)

Of the 1631 white prisoners admitted during the same period, there were 73 deaths, or about 4½ per cent. of the whole number; the per-centage in different years varying as in the case of the coloured. Thus, in 1830, the per-centage is 4.19; in 1831, 4.18; in 1832, 1.44; in 1833, 1.11; in 1834, .80; in 1835, 1.26; in 1836, .99; 1837, 3.00, &c., &c. The average mortality of the white population of the city and liberties, during the decennial period above referred to, was 2.37.

The mortality above indicated would be somewhat increased by taking into account the number of pardons granted on account of ill-health within this period; but, inasmuch as we have no means of arriving at this, we shall exclude it from the calculation. It may be stated, however, that to the close of the year 1848, 278 prisoners had partaken of executive clemency.

* Read by Dr. Parrish at a meeting of the College of Physicians of Philadelphia, Sept. 2nd, 1851.

At the county prison at Moyamensing, built and conducted upon the same plan, we have the following general results:—Taking, for the sake of convenience, a period of thirteen years, viz., from 1835 to the close of the year 1848, we find that 2818 prisoners were sentenced to separate confinement in the convict department of this establishment; of these 1526 were white, and 1392 coloured. These prisoners are from the city population, and are of course in a less vigorous state of health on admission than those from the country; but their sentences are, as a general rule, short,—in a large proportion of cases not exceeding a year, and a majority of them between three and nine months.

Of the 1526 whites, 37 died, or at the rate of about 25 in the 1000, or $2\frac{1}{2}$ per cent. Of the 1392 coloured, 118 died, being a fraction over 80 in the 1000, or $8\frac{1}{2}$ per cent. It will be seen that the same disparity exists here in the mortality between the white and coloured; though the average for both colours falls far below that of the State institution; a fact which forcibly illustrates the comparative influence of long and short sentences in separate prisons.

In both institutions, every attention is paid to the diet, clothing, and medical supervision of the prisoners; the construction of the buildings, and the general course of discipline are the same in each, except that in the county prison the cells are not provided with exercise yards, and the intercourse between the prisoners through the windows opening out upon the main yard, and by other means, is much more easy than in the State institution.

With this summary view of the condition of the prisons in our own vicinity, let us turn for a few moments to the state of things in England, as reported to us in the paper referred to at the opening of this communication.

It has been remarked, that the British government has, within a few years past, adopted the plan of separate confinement in her penal institutions; and the experience thus acquired has frequently been quoted in this country as confirmatory of the safety and efficiency of this system.

It must be borne in mind, however, that the separate system in England is a far different thing from the Pennsylvania system. It is, in fact, divested of some features which are considered fundamental with us. Let me briefly explain the difference.

Separate confinement in England is but a part of a general scheme of management and disposal of convicts, and not, as with us, the only means of satisfying the requisitions of the law. It is, in fact, a probationary stage, intended for the moral training of the prisoner, prior to his entering into associated labour on the public works, or in the colonies. The maximum period to which separate confinement is carried there is eighteen months, while the sentences rarely exceed a year, and in many instances are not over six months. Beyond these periods it is not considered safe by some of the best authorities. Thus, Major J. Jebb, Inspector-General of Prisons, remarks, in one of his recent reports to Parliament: "My own independent conclusion, founded on close observation of the system carried out at Portsmouth, Wakefield, and other prisons, is that separate confinement, with very few exceptional cases, and with ordinary precautions, may be safely and generally adopted for periods extending from six to twelve months, or rather more. Beyond twelve months I think it requires greater care and watchfulness than would perhaps be insured under ordinary circumstances, and there are grounds for believing that it is neither necessary nor desirable to extend it." (See first Report on Portland Prison, &c., by Lieut.-Col. Jebb, presented to both houses of Parliament by order of her Majesty, 1850.)

Pentonville and Millbank are the two most extensive separate prisons in England; they are of modern construction, and are said to be admirably arranged and managed. The former went into operation in 1842, and cost the government 85,000*l.*, or 162*l.* per cell; it is capable of accommodating 500.

The latter has been built since, at a cost of half a million sterling, with accommodations for 1100 convicts. Besides these, there are five or six smaller institutions, in different parts of the kingdom, containing in all, according to the reports for 1850, 2686 persons who were undergoing the probationary period of separate confinement.

Dr. Winslow estimates the known registered mortality of these prisons at 19 in the 1000, exclusive of pardons for ill-health, which, being included, would bring it up to $23\frac{4}{5}$ per 1000, or a fraction over two per cent.

The diseases which are the main outlets of life are, as with us, scrofula and consumption. At Pentonville, the mortality, since its opening in 1842, has been $13\frac{3}{4}$ per 1000; at Millbank, $18\frac{1}{2}$ per 1000. At the Pentonville establishment, the prisoners are picked men, and none are admitted who have not been pronounced by the physician in the best mental and bodily health. Of the first 1000 thus admitted, 11 died of consumption, and 14 were pardoned for having contracted the disease, which would make the rate of mortality from this disease alone $2\frac{1}{2}$ per cent. amongst men selected for their physical vigour. The highest ratio of mortality in any one of the English separate prisons is, according to Dr. Winslow, 41 deaths to the 1000; this is at Reading, which contained in 1850 only 40 convicts.

It will now be seen, that the mortality of the separate plan prisons in England, although regarded as high, falls considerably below that of the Philadelphia establishments.

Thus we have had at Cherry Hill at the rate of 90 deaths to the 1000 prisoners, and at Moyamensing about 52 to 1000, including the deaths of coloured prisoners; comparing the white mortality alone, the disproportion is much less. Thus, we have at Cherry Hill 45 to 1000, and at Moyamensing about 25 to the 1000, exclusive of the pardons, the latter being but little above the registered mortality of the English prisons, stating it at $23\frac{4}{5}$, and the former exceeding but little the mortality of the prison at Reading, 41 in the 1000.

Did time permit, the causes of this disproportion in the deaths in the English prisons and our own might, I think, be satisfactorily explained; and in connection therewith some cogent arguments furnished in favour of a modification of the system now in operation in both countries; but I fear that the patience of the College is already wearied, and I must therefore ask their indulgence for a few moments while I present another branch of this inquiry of still greater importance.

The tendency to separate confinement to induce insanity has been constantly alleged as a most serious objection to its adoption, while the more ardent friends of the system have either totally denied this tendency, or have considered it a contingent circumstance, and not a necessary accompaniment of its proper administration.

Examining this question as it is presented to us in the medical reports, and collating the results, we shall find no difficulty in arriving at the truth.

Dr. Winslow states the cases of mental disorder in the Pentonville prison, between January 1, 1843, and June 30, 1850, at 42 out of 3050 prisoners, being 13.7 per 1000, or $5\frac{3}{5}$ greater than the average in the community; at which rate, were it general, England would have, in 1851, near upon 50,000 male pauper lunatics within her borders!

In Millbank, 34 cases are reported out of 18,520 adults, and 9 cases in 2024 juveniles. And at the meeting of the society at which these statements were made, Dr. Webster stated that, within the last three years, 20 insane prisoners had been sent to the Bethlem Hospital from the Penitentiary at Millbank, and 12 from Pentonville, thus making 32 lunatic patients from these establishments.

In Portland prison, but 5 cases of insanity are reported in 1450 prisoners, or about $3\frac{1}{2}$ per 1000, being one per 1000 above the average. This is an establishment recently opened in the sea-coast of Portland, for the reception of prisoners who have been undergoing separate confinement; and who are here subjected to a course of moral discipline and industrial training, in associated labour during the day, with separation during the night and at meal-times, prior to their final deportation to a penal colony. During the progress of labour on the public works (which is always carried on under the immediate superintendence of the prison officers), the prisoners are allowed to converse to such an extent as not to interrupt the progress of their work. They are also stimulated to good conduct by becoming eligible for release from penal discipline, and embarkation for the colonies, at an earlier period than their sentences would indicate, and by the prospect of procuring on their arrival constant employment at good wages, with the ultimate privilege of being joined by their families in their new home.

The conclusion reached by Dr. Winslow, in view of these facts, is unfavourable to the opinion that the separate or solitary system, even in the modified and restricted form adopted in England, is innocuous to the mind and body, although he hopes that some modification may yet be devised which will relieve it of the well-founded objections which now hold against it.

The statistics furnished by the prisons at Philadelphia bearing upon the question of insanity, are much more full and conclusive than those from abroad; and I ask the attention of the College to them as affording matter for serious reflection and energetic action on the part of those who regard the just and merciful administration of penal law as amongst the highest objects of civil government.

A full account of all the cases of insanity occurring at the Eastern State Penitentiary has not, up to the present time, been prepared; although there has been, in each year, a tabular statement of the number of cases occurring within the year, with the colour, age, sex, health on admission, hereditary tendency, period of imprisonment at which the attack occurred, the form of the disease, and the result.

It is established, by carefully drawn up tabular statements, that 55 cases of insanity originated in the Penitentiary during a period of six years, with an average population of about 300 prisoners. These occurred at various periods of confinement, but it is worthy of remark that a large proportion of them *were developed in prisoners under long sentences.* Thus 36 of the 55 cases were in prisoners sentenced for more than two years, 12 for two years, and 6 for terms between one and two years, and one for six months.*

This fact is exceedingly interesting when viewed in connection with the observations made in the English prisons, and affords conclusive evidence of the tendency of long sentences to produce insanity in separate plan prisons.

In addition to this statement, it may be added that there are at the present time 43 insane prisoners within the walls at Cherry Hill; out of a population of 300, 30 of these cases have been developed in the institution: the other 13 were more or less insane on admission, although 10 of them were convicted of crime,. and sent there as convicts. Of these 43, 28 are white, and 15 coloured; 41 males and 2 females.

All of these cases are recorded as insane, and are so regarded by the medical officer, and by the overseers in charge of them. Dr. Given, the late physician of the penitentiary, is certainly competent authority on this point. A residence of two years as medical assistant to Dr. Kirkbride, and of seven years at the Penitentiary, besides a high reputation for the conscientiousness and accuracy of his medical opinions, give to his judgment a peculiar value. The cavils of non-professional partisans, however well meaning and sincere may be their desire to advance their peculiar views, can weigh nothing against the testimony of so careful and enlightened a medical observer.

* We regret to be obliged to omit the admirable tabular statement accompanying the article.

Many of the cases of insanity occur in convicts of slender capacity, whose education and mental resources are limited, and who speedily succumb when deprived of society. They are commonly ushered in by frightful hallucinations, such as imaginary sounds outside of the cell; conversation between enemies conspiring to do them injury, with constant apprehension of murder or destruction: then comes on loss of sleep, with its attendant nervous excitement; and if not arrested in this stage by appropriate treatment, more confirmed mania, or dementia follows.

Suspected poison in the food, and consequent refusal to eat, is another common form of hallucination. One prisoner, who is regularly at work, has, for a long time, supposed himself the intended victim of a conspiracy out of doors, and although he is locked in his cell with double doors, front and back, he regularly barricades his doors at night, to keep out his tormentors.

It may be stated farther, on the authority of Dr. Given, confirmed by the testimony of the warden, and of the overseers appointed by him, that the present number of the insane is not excessive; but that for a number of years past, it has been about in the proportion here stated. A majority of the officers place the ratio of insane at 10 per cent., while the lowest estimate is 7 per cent. Admitting it to be 8 per cent., we should have 80 cases of insanity for every 1000 prisoners, or over 200 cases for the 2549 prisoners admitted to the close of the year 1849; exclusive of a large class sent to the institution with their minds more or less diseased.

This estimate does not rest upon vague assertion, or upon the clamour of the opponents of the system; it comes from men deeply interested in the welfare of the institution over which they watch, many of whom have been connected with it for a long series of years, and are thoroughly conversant with its practical working.

In the county prison, the number of insane reported falls far below that of the State institution, and approaches nearer to the British prisons. A statistical table of all the cases occurring between the years 1835 and 1849 gives us 23 cases from 2815 prisoners, and of these, 14 are said to have been more or less insane on admission, leaving but nine cases originating in this prison; a number considerably below Pentonville, above Millbank, and nearly equal to Portland. This striking difference between the State and county prison may, I think, be explained on the same grounds which have heretofore been assumed when speaking of their relative mortality, the difference in the length of sentences being the most important element in the consideration.

From the above comparison of the mortality and insanity of our separate plan prisons with those in England, it will be seen that the State prison at Cherry Hill, where the experiment of separation was first commenced, and where it has been vigorously carried out for more than twenty years, has been the most severely scourged by those disorders which medical experience has proved to follow rigid confinement in close and darkened rooms, without the requisite amount of exercise in the open air, and sun-light. While it is equally evident that the abrogation of the social principle, continued for lengthened periods as practised here, inflicts upon a certain class of minds serious and oftentimes irreparable mischief. The extent of these evils can only be realized by looking through the entire history of this institution, and computing the mortality and insanity from the whole number of persons subjected to its discipline.

It is also observed that in the separate plan prisons of England, where the sentences rarely exceed a year, and in which all the modern improvements of construction have been introduced at an enormous expense, the mortality and insanity, although comparatively trifling, have still been sufficiently high to attract the notice of distinguished medical men, and to induce them to doubt the propriety and humanity of the plan.

This is not the time or place to enter into an examination of the healthfulness of other prisons, as compared with those to which our attention has been directed, or to discuss the relative merits of the plans of imprisonment now in vogue. My object is not to advocate any particular system, but rather to show that an exclusive devotion to one idea on this subject may lead to serious consequences, both as it affects individuals and the State. When we consider the great variety in the physical and mental constitution of man, the many diverse circumstances by which his actions are influenced, and the various grades of moral depravity which mark his departure from a virtuous life, it would seem reasonable that, in graduating punishment, some regard should be had to these differences; and that one invariable method should not be applied to all. While, therefore, we would not abandon the separate principle as applied for *limited periods* to the generality of convicts, we should be equally averse to applying it to those whose physical and mental constitution were proved, by observation, to be peculiarly obnoxious to its influence.

To continue such a course would be visiting calamities upon these erring and rebellious subjects of the State which the law never intended, and from which humanity revolts. What more cruel penalty can be inflicted on a fellow-being than the dethronement of his reason!—to be doomed to a life of sorrow and privation—the creature of sudden impulse, or the passive and imbecile instrument of another's will! What hope of steady and consistent conduct can light the path of those whose minds have been unhinged by the very means taken to enlighten and reform them?

These are questions which naturally arise in view of the facts here presented; they are not the offspring of a sickly sentimentality, or of a morbid sympathy for criminals; they affect alike the honour and reputation of the commonwealth, and the best interests of those who have forfeited their liberty, by violating its laws.

In the remarks here made, I must distinctly disclaim any feeling of opposition to the institutions more especially brought under review. Their management and discipline are, I believe, in accordance with the laws, and those placed in charge of them appear to be generally imbued with a spirit of humanity in carrying out the trust committed to them. That this is the case at Cherry Hill, frequent personal observation for some years past as a visitor and member of the Prison Society, has fully convinced me. I doubt, indeed, if there is a prison in the country supplied with a more intelligent and humane body of officers than this; and none certainly where a greater degree of order, decorum, and good feeling prevails.

There is also, on the part of the chief officers of the institution, no desire to suppress inquiry into the effects of their discipline upon life and health, or to retard such improvements and modifications in the mode of punishment in operation there as enlightened experience may suggest.

I hope the College will excuse me for taking up so much of their time in the examination of a subject which may be regarded by some as not strictly within our province to discuss. I know that there is a feeling abroad opposed to all efforts tending to the farther amelioration of the criminal code, and it is even contended that, by making prisons too comfortable, and their discipline too mild, a direct bounty will be conferred upon crime, and thus one grand check to the evil passions and designs of bad men will be removed. But I trust that, as physicians, desirous of the establishment of truth upon a question of great public moment, no such considerations will deter us from investigations into the effects of the various methods of punishment upon the minds and bodies of the inmates of prisons, and, if we find suffering and disease beyond the limits which the law contemplates or which humanity sanctions, that our voices at least will be raised for their correction.

